# Nursing Practice Environments in Hospitals: A Comparative Study between Portugal and Brazil

**DOI:** 10.3390/nursrep14040212

**Published:** 2024-10-09

**Authors:** Olga Maria Pimenta Lopes Ribeiro, Alessandro Rodrigues Perondi, Jane Tavares Gomes, João Miguel Almeida Ventura-Silva, Marlene Patrícia Ribeiro, Susana Filipa Mendes de Castro, Tânia Dionísia Ferreira Oliveira, Letícia de Lima Trindade

**Affiliations:** 1 Nursing School of Porto, 4200-072 Porto, Portugal; 2 Center for Health Technology and Services Research (CINTESIS@RISE), 4200-450 Porto, Portugal; joao.ventura@essnortecvp.pt; 3 Departament of Nursing, Paranaense University, Paraná 87502-210, Brazil; alessandroperondi@prof.unipar.br; 4 Southwest Regional Hospital Walter Alberto Pecoits, Paraná 85601-839, Brazil; 5 Departament of Nursing, Santa Catarina State University, Florianópolis 88035-901, Brazil; jane.tavares11@hotmail.com (J.T.G.); letrindade@hotmail.com (L.d.L.T.); 6 Portuguese Red Cross Northern School of Health, 3720-126 Oliveira de Azeméis, Portugal; 7 Abel Salazar Institute of Biomedical Sciences, University of Porto, 4050-313 Porto, Portugal; ribeiro.marlene.27@gmail.com (M.P.R.); enf.susanacastro@gmail.com (S.F.M.d.C.); taniadionisia@gmail.com (T.D.F.O.); 8 Tâmega and Sousa Local Health Unit, 4560-136 Penafiel, Portugal; 9 Portuguese Oncology Institute of Porto Francisco Gentil, 4200-072 Porto, Portugal; 10 Alto Ave Local Health Unit, 4835-044 Guimarães, Portugal

**Keywords:** hospitals, nursing, professional practice, work environment

## Abstract

**Background:** Assessing the elements of nursing practice environments is crucial, as investing in their improvement will enhance outcomes for nurses, patients, and organizations. Moreover, comparing practice environments from different countries improves the definition of cross-cutting guidelines that can be applied in various contexts. Thus, this study aims to evaluate nursing practice environments in hospitals in Portugal and Brazil. **Methods:** A multicenter and cross-sectional study was conducted in eight Portuguese and eight Brazilian hospitals. Five hundred eighty-two nurses completed a survey regarding their sociodemographic and professional attributes, as well as the Scale for the Environments Evaluation of Professional Nursing Practice. The data were subjected to comparative analyses between the two countries. We adhered to ethical requirements in both participating countries. **Results:** In Brazil, the Structure, Process, and Outcome components were considered favorable to the quality of care and well-being of nurses. In Portugal, nurses considered the Structure and Outcome components favorable and the Process component very favorable. Statistically significant differences were found between the two countries in several dimensions of the three subscales. The Structure and Outcome components scored significantly better in hospitals in Brazil, and the Process component scored better in Portuguese hospitals. **Conclusions:** These conclusions underscore the urgent need for investment in continuous training and a culture of evaluation that promotes continuous improvement. Additionally, promoting the involvement and participation of nurses could simultaneously contribute to the development of more sustainable health systems.

## 1. Introduction

From Lake’s perspective, the nursing practice environment refers to the organizational characteristics of the work context that can favor or limit nurses’ professional practice, producing results for patients, nurses, and health organizations [1].

Since 2007, the International Council of Nurses (ICN) has released recommendations highlighting that nursing practice environments must be based on innovative political structures that promote nurses’ well-being, professional satisfaction, safety and quality of care, and excellent organizational performance [2]. Studies have revealed that favorable practice environments are positively associated with transformational leadership styles, quality and safety in care, structural and human resources adequacy, adjusted workloads, professional satisfaction, and low nurse turnover [3,4,5,6]. In addition, a positive environment contributes to nurse autonomy and professional recognition, promotes efficient leadership, ensures safe staffing, and enhances teamwork and motivation [7,8,9].

The most common factors that positively influence nursing practice environments are communication, collaboration, teamwork, meaningful recognition, professional autonomy, effective decision-making, appropriate staffing, physical and psychological safety, and authentic leadership [6,9].

In the past decade, there has been a growing concern about investing in nursing practice environments. Nursing professionals faced challenges exacerbated by the COVID-19 pandemic, especially in hospital settings. Limited resources and pre-existing vulnerabilities, intensified by the pandemic, have led to increased demands and efforts by nurses to maintain safe practice environments capable of ensuring the quality care and the professionals’ well-being [10,11,12,13,14].

In 2024, the ICN once again reinforced the need for advanced strategies for recruiting and retaining nurses, given the global shortage of these professionals degraded by the pandemic [15]. In the national and international context, some contributions have emerged to leverage the promotion of positive practice environments. Providing guiding resources for its implementation, creating assessment instruments for these environments, and identifying associated indicators have helped improve practice environments [16,17]. Therefore, the Scale for the Environments Evaluation of Professional Nursing Practice (SEE-Nursing Practice) has been an added value [18].

The SEE-Nursing Practice was developed and validated in 2021 to evaluate practice environments based on three components: Structure, Process, and Outcome [18,19]. The Structure component portrays the organizational elements that allow nurses to practice their profession. The Process component includes factors related to the performance of nursing care activities. The Outcome component evaluates positive or negative changes in care, patients, and nurses [11,18,20].

It is imperative to assess the professional practice settings of institutions and services because the opportunity to enhance the nursing practice environment can only arise from an understanding of reality [18,21,22]. The significance of this phenomenon becomes more pronounced when considering with the realities of nursing practice environments in the national and international hospital setting [8]. The variations in the health system, professional legal framework, training, and socioeconomic conditions among countries can impact practice environments and outcomes, demanding further study. Analyzing this diversity is essential in establishing guidelines that can help promote more positive nursing practice environments, regardless of the country.

This viewpoint supported the execution of a multicenter study designed to assess the nursing professional practice environments in Portuguese and Brazilian hospitals. The choice of these two countries is based on their joint investment in this area and the relevance of identifying factors that, in any given situation, address the primary concerns of nurses in both countries: enhancing working conditions and recognizing the essential role of these professionals in their respective health systems.

The nursing practice environments in these two countries share the common challenge of being frequently affected by structural and financial limitations, which can compromise the quality and safety of care, as well as the well-being of professionals [23]. One of the most distinct aspects of the practice environments in these countries concerns the organizational methodology of nursing care. The most common work method used by nurses in hospitals in Brazil is the team-based method, with a division of responsibilities among professionals with different levels of education (nurses, nursing technicians, and nursing assistants), promoting collaborative and coordinated care. Each team member has specific roles, with the nurse being responsible for planning, supervising, and evaluating the care provided [13].

In the case of hospitals in Portugal, the nursing team is exclusively composed of nurses, with a strong focus on the nursing decision-making process, both in the autonomous and interdependent domains of the profession. The most common work method is the individual method, in which the same nurses are responsible for the design and provision of care to meet all the patients’ needs [24].

In light of the above, studies of this nature align with the Sustainable Development Goals (SDGs), as they provide elements to improve the quality of care. They potentially lead to health and well-being (SDG 3), decent work and economic growth (SDG 8), and reduced inequalities (SDG 10) [25] based on conclusions guiding best practices that help to inform policies and strategies to strengthen health systems globally because they face new care challenges caused by increasingly complex health problems and changes in the population’s expectations.

## 2. Materials and Methods

### 2.1. Design

This is a multicenter, cross-sectional, quantitative study. We used the Strengthening the Reporting of Observational Studies in Epidemiology (STROBE^®^) tool to ensure the methodological accuracy, quality, and transparency of the scientific writing [26].

### 2.2. Setting and Sample

The research was conducted in 16 hospitals, eight in Portugal’s northern region and eight in Brazil’s southern region. These hospitals were deliberately selected because they are all public reference institutions of medium and high complexity and are close to the research institutions. It is important to highlight that none of these institutions are university hospitals.

To select our sample, we applied a non-probabilistic convenience sampling technique. The inclusion criteria were as follows: (a) being a nurse working in the hospitals under study, particularly in the departments of Medicine, Surgery, Emergency and Intensive Care, Psychiatry and Mental Health, and Woman and Child Department; (b) having at least six months of experience in the unit; and (c) being in professional practice during the data collection period, i.e., not on vacation or extended leave.

Following this, considering a heterogeneity of 50%, a 95% confidence interval, and a 5% sampling error, a minimum sample size of 282 participants was obtained from a population of 1059 nurses in Portugal. Applying the same sampling criteria in Brazil, a minimum sample size of 232 participants was obtained from a population of 583 nurses. It is important to keep in mind that the minimum number of participants was proportionally stratified across each hospital where the study was conducted. Given the adherence of nurses who met the inclusion criteria, the final sample comprised 291 Portuguese nurses and 291 Brazilian nurses, totaling 582 participants who completed all the questions in the data collection instrument.

### 2.3. Data Collection

Data collection occurred from July to November 2021 during on-site visits to hospital institutions. We invited the nurses who met the inclusion criteria to take part in the research. We informed the nurses about the study’s objective and the necessity of expressing their consent. We then asked them to sign the informed consent form and provided them a printed questionnaire. The questionnaire comprised two sections. The first part collected information on sociodemographic and professional characteristics (age, gender, marital status, education, work context, time of professional practice in the profession, and time of professional practice in the current service/unit).

The second part contained the Scale for the Environments Evaluation of Professional Nursing Practice (SEE-Nursing Practice) [18], which was linguistically adapted to Brazilian Portuguese for this study [21]. The SEE-Nursing Practice consists of three subscales: the SEE-Nursing Practice—Structure, the SEE-Nursing Practice—Process, and the SEE-Nursing Practice—Outcome [18].

The SEE-Nursing Practice—Structure is made up of 43 items, divided into six dimensions: 1—People management and service leadership (12 items); 2—Physical environment and conditions for service running (13 items); 3—Nurses’ participation and involvement in policies, strategies, and running the institution (8 items); 4—Institutional policy for professional qualification (3 items); 5—Organization and guidance of nursing practice (4 items); and 6—Quality and safety of nursing care (3 items).

The SEE-Nursing Practice—Process consists of 37 items, distributed across six dimensions: 1—Collaboration and teamwork (9 items); 2—Strategies for ensuring quality in professional practice (7 items); 3—Autonomous practices in professional practice (7 items); 4—Care planning, evaluation and continuity (6 items); 5—Theoretical and legal support of professional practice (4 items); and 6—Interdependent practices in professional practice (4 items).

The SEE-Nursing Practice—Outcome includes 13 items divided into two dimensions: 1—Systematic assessment of nursing care and indicators (7 items); and 2—Systematic assessment of nurses’ performance and supervision (6 items).

In SEE-Nursing Practice, the response is measured for each item on a Likert-type scale with five options: one corresponds to “never”, two to “rarely”, three to “sometimes”, four to “very often”, and five to “always”. It is important to highlight that the higher the score on the SEE-Nursing Practice is, the more favorable the professional nursing practice environment is for the quality of care and the well-being of nurses. According to the authors’ guidelines, the following criteria should be established to evaluate the results: a score < 35%—component/dimension of the environment of professional nursing practice that is not favorable to the quality of care and the well-being of nurses; a score between 35% and 55%—component/dimension of the professional nursing practice environment moderately favorable to the quality of care and the well-being of nurses; a score between 55% and 75%—component/dimension of the professional nursing practice environment favorable to the quality of care and the well-being of nurses; and a score >75%—component/dimension of the professional nursing practice environment very favorable to quality of care and the well-being of nurses [12].

Regarding the internal consistency of the instrument, the Cronbach’s Alpha values of the SEE-Nursing Practice components concerning Portugal were 0.957 for Structure, 0.936 for Process, and 0.926 for Outcome. These values were overall higher than those of the previous study [18]. The internal consistency of the Brazilian version obtained a Cronbach’s Alpha coefficient for the subscales of the SEE-Nursing Practice—Structure, Process, and Outcome of 0.956, 0.929, and 0.937, respectively [21].

### 2.4. Data Analysis

We manually entered the quantitative data into a Microsoft Excel spreadsheet and then transferred the data to IBM Statistical Package for the Social Sciences (SPSS), version 26.0 (Armonk, New York, NY, USA). First, we applied descriptive statistics to analyze the categorical and numerical variables. We used absolute and relative frequencies and the chi-squared test (X2) to analyze differences in categorical sociodemographic and professional variables between the two countries. For numerical sociodemographic and professional variables, we used the mean, standard deviation, and Mann–Whitney test to analyze differences between the two countries.

Regarding nursing practice environments, to compare the analysis of the results between the two countries, we initially tested the normality of the scores for each dimension and subscale using the Shapiro–Wilk and Lilliefors (Kolmogorov–Smirnov) tests. As both tests rejected normality, we used the Mann–Whitney test to compare the two countries.

All analyses used a 0.05 significance level.

### 2.5. Ethical Considerations

The research was approved by the Ethics Committee in both countries and authorized in all hospitals in Brazil (process no. 4.722.300) and Portugal (process no. 104/21). All participants signed the Free and Informed Consent Form, and the research team completed a training process to conduct the study in both countries, standardizing theoretical-methodological conduct.

## 3. Results

This study had 582 nurses, including 291 from Portugal and 291 from Brazil. Their sociodemographic and professional characteristics are explained in Table 1.

Concerning the Structure component assessed using the SEE-Nursing Practice—Structure subscale, for the dimensions “People management and service leadership” (Dimension 1), “Physical environment and conditions for service running” (Dimension 2), “Nurses’ participation and involvement in policies, strategies, and running the institution” (Dimension 3), and “Organization and guidance of nursing practice” (Dimension 5), as well as for the Structure subscale itself, it was found that the average frequency was lower in Portugal. In the dimensions “Institutional policy for professional qualification” (Dimension 4) and “Quality and safety of nursing care” (Dimension 6), it is assumed that the average frequency was the same in both countries.

The dimension “Quality and safety of nursing care” (Dimension 6) obtained the best qualification in both countries, and in Brazil, it was considered very favorable to the quality of care and the well-being of nurses. The remaining five dimensions were considered favorable to the quality of care and the well-being of nurses in both countries. Regarding the worst scored dimensions, in Portugal, it was “Nurses’ participation and involvement in policies, strategies and running the institution” (Dimension 3), and in Brazil, it was “Institutional policy for professional qualification” (Dimension 4). In both countries, the Structure component was considered favorable to the quality of care and the well-being of nurses.

Table 2 shows the mean, standard deviation, score of “Quality of Nursing Practice Environment” for each dimension of the Structure subscale, and the *p*-value of the Mann–Whitney test.

In the Process component, which was assessed using the SEE-Nursing Practice—Process subscale, for the dimensions “Strategies for ensuring quality in professional practice” (Dimension 2), “Care planning, evaluation, and continuity” (Dimension 4), “Theoretical and legal support of professional practice” (Dimension 5), and “Interdependent practices in professional practice” (Dimension 6), as well as for the Process subscale itself, it is concluded that the average frequency was lower in Brazil. Concerning the dimensions “Collaboration and teamwork” (Dimension 1) and “Autonomous practices in professional practice” (Dimension 3), it is assumed that the average frequency was the same in both countries.

The dimension “Theoretical and legal support of professional practice” (Dimension 5) was the one that obtained the best qualification in both countries and was the only dimension in Brazil that was considered very favorable to the quality of care and the well-being of nurses. In Portugal, in addition to “Theoretical and legal support of professional practice” (Dimension 5), the dimensions “Care planning, evaluation, and continuity” (Dimension 4) and “Interdependent practices in professional practice” (Dimension 6) were scored as very favorable to the quality of care and the well-being of nurses.

Regarding the worst-scored dimensions, in Portugal, it was “Strategies for ensuring quality in professional practice” (Dimension 2), and in Brazil, it was “Interdependent practices in professional practice” (Dimension 6). Examining the Process component globally, in Portugal, it was considered very favorable to the quality of care and the well-being of nurses, and in Brazil, it was favorable to the quality of care and the well-being of nurses.

Table 3 presents the mean, standard deviation, and score of “Quality of the Nursing Practice Environment” for each dimension of the Process subscale, as well as the *p*-value of the Mann–Whitney test.

Concerning the Outcome component, which was evaluated using the SEE-Nursing Practice subscale—Outcome, for the dimensions “Systematic assessment of nursing care and indicators” (Dimension 1) and “Systematic assessment of nurses’ performance and supervision” (Dimension 2), as well as in the Outcome subscale itself, it was found that the average frequency was lower in Portugal. The dimension “Systematic assessment of nurses’ performance and supervision” was the worst qualified in both countries. In Portugal, it was considered moderately favorable to the quality of care and the well-being of nurses.

Overall, the Outcome component was considered favorable to the quality of care and the well-being of nurses in both countries (Table 4).

## 4. Discussion

This study evaluated Portuguese and Brazilian nurses’ perceptions of nursing practice environments in the hospital context.

Regarding sociodemographic characteristics, it was found that nurses in Brazilian hospitals had a lower average age than nurses in Portuguese hospitals. The average length of professional experience and professional experience within services were also lower among nurses in Brazil. Another relevant conclusion concerns professional qualifications, where a higher number of participants with a master’s or doctoral degree were found among nurses in Brazilian hospitals.

Indeed, when the report “State of the world’s Nursing 2020: Investing in Education, Jobs and Leadership” was published by the World Health Organization [27], Oliveira et al. [28] reported that the nursing workforce in Brazil was considered relatively young and that 38% of nurses were under 35 years of age, being at the beginning of their career. Furthermore, the authors added that the results found for nurses over 55 years old were also different from those found in other countries in the Americas and Europe, as there was a lower percentage of nurses in this age group in Brazil [28].

Concerning the qualification of professionals, in Portugal, the professional development model for nurses includes three careers: the general care nurse, the specialist nurse in an area of expertise in nursing, and the nurse manager [29]. Until 2022, it was possible to undertake postgraduate training in a nursing specialty area, granting the title of specialist in this specialty area and access to a career as a specialist nurse without completing the master’s degree. In fact, for nurses who intended to work in hospitals, there was no monetary recognition for the master’s degree. Currently, in Portugal, the courses leading to the degree of the title of specialist in any area of nursing specialty and access to the respective career are master’s courses. Thus, there is expected to be an increase in the number of nurses with a master’s degree working in hospitals in Portugal in the coming years [30].

Observing nursing practice environments, the Structure component found that conditions were favorable to the quality of care and the well-being of nurses in hospital institutions in both countries, except for one dimension. The lower scores in Portuguese institutions refer to the apprehension already expressed by nurses that institutions could gradually reduce the investment made during the pandemic [31]. In a study previously carried out in Portugal, researchers confirmed a positive impact of the pandemic on nursing practice environments, particularly in the Structure component, which, from their perspective, indicated that the institution’s investment in areas that were previously more fragile had an impact on better environment [32]. A study in China also showed that the pandemic was associated with improved nursing practice environments [33].

The problem is that, along with the investment made throughout the pandemic, it is essential to maintain actions to promote positive nursing practice environments [11,31]. The lack of continuity in investment in maintaining and improving working conditions can have negative repercussions [32]. Studies conducted by the American Association of Critical-Care Nurses warn that actions to improve nursing practice environments are urgently needed; however, addressing the team without investing in the work environment is ineffective due to the symbiotic nature of their relationship [6]. According to the authors, without improvements in the practice environment, nurses will continue to leave institutions and even the profession itself and search for more meaningful, rewarding, and sustainable work. Additionally, it is worth noticing that more experienced nurses’ express fatigue and demotivation related to many years of advocating for better working conditions.

Fortunately, data from the studies conducted also show that an active and continuous focus on the work environment makes a difference, and overall, outcomes for patients, professionals, and the organizations themselves are improved when the environment is addressed [6]. In fact, regardless of some weaknesses encountered in this study, the institutions’ concern with defining guidelines to maintain the quality and safety of nursing care was reflected in the fact that the “Quality and safety of nursing care” dimension obtained the best qualification in both countries.

The worst score in Portugal in the dimension “Nurses’ participation and involvement in policies, strategies and running the institution” and in Brazil in the dimension “Institutional policy for professional qualification” is in line with other studies in both countries. In Portugal’s case, in recent years, all studies carried out report the lack of participation and involvement of nurses in the definition of institutional policies, with a negative impact on their satisfaction and involvement [11,32]. In research carried out in Brazil on working environments in hospitals during the pandemic, in terms of unfavorable aspects, nurses highlighted their minimal participation in decisions [13].

The results obtained in the Structure component corroborate the idea that investing in nurses’ participation, involvement, and professional qualification is necessary [23]. From the authors’ perspective [11,32,34], these aspects are strongly dependent on the management strategies and leadership styles in force in the institutions.

In the Process component, for five of the six dimensions of this subscale, the average scores were higher in Portuguese institutions. The high average score in the “Care planning, evaluation, and continuity” dimension reflects the investment made in Portugal in the conception and provision of nursing care and nursing information systems [35].

Such results may also be related to the fact that, in Portugal, the training process is exclusively linked to higher education, where there has been a focus on the clinical decision-making process, contributing significantly to the development of the autonomous and interdependent components of the profession.

Regarding autonomous and interdependent practices, although the scores indicate that these dimensions are favorable to the quality of care and the well-being of nurses in both countries, there are opportunities for improvement. Authors point out that the nurses’ professional practice model in the hospital context is influenced by theory and practice and that task-centered care provision favors fragmented and automated practices that make it difficult to move away from the biomedical model [36]. In both countries, the predominance of the technical and fragmenting model in nursing training, to which Saraiva et al. [37] refer, can justify the results. Despite the most critical results in Brazil, researchers warn that Portuguese nursing is also experiencing a moment of transition between a practice still inspired by the biomedical model and a practice already visible, with a remarkable influence from the theoretical references of the nursing discipline [36]. A performance more aligned with these disciplinary references will enhance the development of autonomous practices [38], distancing nurses from the trajectory of reproducing medical, hospital-centered, and technical knowledge, which is widely recognized as characteristic of the biomedical model [37,38].

The average score of “Theoretical and legal support of professional practice” in both countries reflects a dimension very favorable to the quality of care and the well-being of nurses. This fact aligns with the historical trajectory of nursing, marked by Florence Nightingale, the pioneer of modern nursing, and reflects ongoing efforts to develop the profession and discipline of nursing worldwide [39]. Each country’s history marks this trajectory uniquely, with regulatory councils, schools, and associations making essential contributions.

Although the profession’s regulatory instruments and theoretical references are decisive for quality and safety, the results for the dimension “Strategies for ensuring quality in professional practice” reveal a need for more significant investment. In prior research conducted in hospitals in Portugal, the authors emphasized the importance of reassessing current practices and aligning actions with established quality standards [40].

In this context, organizational and management strategies are crucial [41]. Moments of sharing knowledge and experiences about customer care along with joint reflection on quality indicators, audits, and care evaluation processes can boost the involvement of all professionals and improve the quality and safety of care provided to people. Supervision of care and investment in a proper safety culture, already previously identified [36], are two of the most relevant strategies to ensure the quality of professional practice, both in terms of the autonomous dimension as an interdependent dimension of the profession.

Finally, concerning the Outcome component, the results for both countries show the need for investments in the systematic evaluation of care, nursing indicators, and the performance and supervision of nursing professionals. The fact that nurses in hospitals in Brazil place emphasis on monitoring indicators may explain the higher score obtained in the Outcome subscale [42,43]. Within the scope of improvement strategies, it is crucial to consider the interaction between supervision and nursing indicators, which can affect the quality of the results of nursing practices. There is a growing debate in the literature about the importance of developing a contextualized model of clinical nursing supervision, guiding the monitoring of indicators, implementing improvement actions according to the results, establishing a proximity response, and facilitating responsible decision-making as well as evidence-based practice, which are fundamental aspects for professional and organizational development and for promoting best practices [44,45]. The existence of a model seems to be the guide for potential changes in behavior and the profession’s identity since supervision directly and significantly influences skills at a personal and professional level.

Although the aim of the study was to evaluate nursing practice environments in hospitals in Portugal and Brazil, the results highlight the urgent need for investment in these areas. Raising awareness among all nursing professionals, particularly institutional leaders and government officials, is essential, as the future of health systems and patient lives depend on it [6].

Regardless of the country, the study’s results point to the development and implementation of interventions, practices, and training programs aimed at improving nursing practice environments in hospital settings [9,46]. These should mainly focus on the participation and involvement of nurses in institutional policies, strategies, and operations, as well as the services where they work. Key areas include promoting professional qualification, ensuring the quality of professional practice in both autonomous and interdependent dimensions, and implementing performance assessment and supervision models that guarantee the quality of care provided and the well-being of nurses.

### Limitations

Despite the relevance of the results, this study has some limitations. First, the use of convenience sampling to present results from a multicenter project does not allow the generalization of the results. The causal relationship cannot be determined, and participants may not be representative of all nurses. Moreover, since the study relied on participants’ self-reporting, it is essential to consider the potential for response bias. Therefore, by meeting the inclusion criteria, multiple nurses may participate in the survey, potentially leading to a biased sample due to interest in the topic. In conclusion, it should be noted that the research was conducted soon after the pandemic outbreak, potentially impacting the results. Nevertheless, the study encourages replication in other contexts to understand whether weaknesses are shared and whether improvement strategies can be generalized and replicated, although adjustable to contexts, with international cooperation being an essential route for research and professional advancements.

## 5. Conclusions

This study allowed Portuguese and Brazilian nurses to evaluate their professional practice environments. The results reveal that the Structure and Outcome components scored significantly better in Brazil, unlike the Process component, which obtained a better score in hospitals in Portugal. The need to invest in nurses’ participation and involvement in defining policies, professional qualification, systematic care evaluations, performance indicator, and supervision is common concern for both countries.

The results from different countries provide essential insights into the contribution of institutions’ middle and top management strategies to improving the dimensions of practice environments, supporting the implementation of changes, and assessing their impact. They provide evidence for developing positive practice environments for patients, professionals, and organizations, contributing to reducing professional stress and burnout, nursing workforce retention, and excellent institutional safety. They suggest that hospitals need support to develop environments that encourage continued training and a culture of evaluation among nurses. Additionally, promoting nurses’ participation and involvement in defining hospital policies is essential, as these aspects lead to more effective and efficient practices, ultimately contributing to more sustainable health systems.

## Figures and Tables

**Table 1:** Sociodemographic and professional characterization of participants from Portugal and Brazil.

Sociodemographic and Professional Characteristics	Portugal	Brazil	*p*-Value
**Gender n (%)**			0.211 *
Female	239 (82.1)	251 (86.3)	
Male	52 (17.9)	40 (13.7)	
**Marital status** n (%)			0.198 *
Single, Divorced, Widower	90 (30.9)	75 (25.8)	
Married/non-marital partnership	201 (69.1)	216 (74.2)	
**Age (years)** Mean; Std. Dev.	40.4; ±9.6	34.2; ±8.4	<0.001 **
**Education** n (%)			<0.001 *
Bachelor’s degree	224 (77.0)	100 (34.4)	
Master’s degree	64 (22.0)	176 (60.5)	
Doctoral degree	3 (1.0)	15 (5.1)	
**Work Department** n (%)			0.103 *
Medicine Department	125 (43.0)	158 (54.3)	
Surgery Department	43 (14.8)	37 (12.7)	
Emergency and Intensive Care Department	57 (19.6)	61 (21.0)	
Psychiatry and Mental Health Department	31 (10.7)	11 (3.8)	
Woman and Child Department	35 (12.0)	24 (8.2)	
**Time of professional practice (years)** Mean; Std. Dev.	17.5; ±9.8	8.0; ±7.1	<0.001 **
**Time of professional practice in the service (years)** Mean; Std. Dev.	9.4; ±8.2	4.9; ±5.6	<0.001 **

* Chi-square test. Significant test (5% significance level). ** Mann–Whitney test. Significant test (5% significance level). Std. Dev.—Standard deviation.

**Table 2:** Comparative results of the Structure component of the professional nursing practice environment in Portugal and Brazil.

			Portugal			Brazil			
Dimensions	Min.	Max.	Mean	Std. Dev.	QNPE %	Mean	Std. Dev.	QNPE %	*p*-Value *
Dimension 1	12	60	42.7	9.6	71.2	44.7	8.4	74.5	0.012 *
Dimension 2	13	65	42.3	8.2	65.1	45.2	8.7	69.5	0.001 *
Dimension 3	8	40	22.6	5.6	56.5	25.2	6.4	63.0	<0.001 *
Dimension 4	3	15	9.0	2.4	60.0	8.5	2.9	56.7	0.077
Dimension 5	4	20	13.7	3.1	68.5	14.7	3.0	73.5	<0.001 *
Dimension 6	3	15	11.2	2.3	74.7	11.3	2.3	75.3	0.581
Structure subscale	43	215	141.5	26.7	65.8	149.6	26.3	69.6	0.001 *

* Mann–Whitney test. Significant test (5% significance level). Min.—Minimum, Max.—Maximum, Std. Dev.—Standard deviation. QNPE—“Quality of the Nursing Practice Environment” Score. Dimension 1—People management and service leadership. Dimension 2—Physical environment and conditions for service running. Dimension 3—Nurses’ participation and involvement in policies, strategies, and running the institution. Dimension 4—Institutional policy for professional qualification. Dimension 5—Organization and guidance of nursing practice. Dimension 6—Quality and safety of nursing care.

**Table 3:** Comparative results of the Process component of the professional nursing practice environment in Portugal and Brazil.

			Portugal			Brazil			
Dimensions	Min.	Max.	Mean	Std. Dev.	QNPE %	Mean	Std. Dev.	QNPE %	*p*-Value *
Dimension 1	9	45	33.7	6.0	74.9	32.8	5.0	72.9	0.058
Dimension 2	7	35	25.2	5.1	72.0	24.0	5.1	68.6	0.003 *
Dimension 3	7	35	26.1	4.3	74.6	26.6	4.3	76.0	0.090
Dimension 4	6	30	23.1	3.9	77.0	22.4	3.6	74.7	0.012 *
Dimension 5	4	20	16.6	2.4	83.0	15.6	2.4	78.0	<0.001 *
Dimension 6	4	20	15.0	2.8	75.0	13.2	2.7	66.0	<0.001 *
Process subscale	37	185	139.8	19.8	75.6	134.6	18.3	72.8	0.001 *

* Mann–Whitney test. Significant test (5% significance level). Min.—Minimum, Max.—Maximum, Std. Dev.—Standard deviation. QNPE—“Quality of the Nursing Practice Environment” Score. Dimension 1—Collaboration and teamwork. Dimension 2—Strategies for ensuring quality in professional practice. Dimension 3—Autonomous practices in professional practice. Dimension 4—Care planning, evaluation, and continuity. Dimension 5—Theoretical and legal support of professional practice. Dimension 6—Interdependent practices in professional practice.

**Table 4:** Comparative results of the Outcome component of the professional nursing practice environment in Portugal and Brazil.

			Portugal			Brazil			
Dimensions	Min.	Max.	Mean	Std. Dev.	QNPE %	Mean	Std. Dev.	QNPE %	*p*-Value *
Dimension 1	7	35	22.7	5.6	64.9	25.0	5.6	71.4	<0.001 *
Dimension 2	6	30	16.3	5.0	54.3	18.8	5.2	62.7	<0.001 *
Outcome subscale	13	65	39.0	9.9	60.0	43.7	10.0	67.2	<0.001 *

* Mann–Whitney test. Significant test (5% significance level). Min.—Minimum, Max.—Maximum, Std. Dev.—Standard deviation. QNPE—“Quality of the Nursing Practice Environment” Score. Dimension 1—Systematic assessment of nursing care and indicators. Dimension 2—Systematic assessment of nurses’ performance and supervision.

## Data Availability

The data that support the results of this study are available from the corresponding author upon reasonable request.

## Public Involvement Statement

There was no public involvement in any aspect of this research.

## Guidelines and Standards Statement

This manuscript was drafted against the STROBE^®^ (Strengthening the Reporting of Observational Studies in Epidemiology) for observational studies research.
